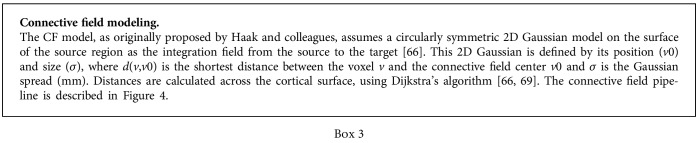# Studying Cortical Plasticity in Ophthalmic and Neurological Disorders: From Stimulus-Driven to Cortical Circuitry Modeling Approaches

**DOI:** 10.1155/2019/2724101

**Published:** 2019-11-03

**Authors:** Joana Carvalho, Remco J. Renken, Frans W. Cornelissen

**Affiliations:** ^1^Laboratory of Experimental Ophthalmology, University Medical Center Groningen, University of Groningen, Groningen, Netherlands; ^2^Cognitive Neuroscience Center, University Medical Center Groningen, University of Groningen, Netherlands

## Abstract

Unsolved questions in computational visual neuroscience research are whether and how neurons and their connecting cortical networks can adapt when normal vision is compromised by a neurodevelopmental disorder or damage to the visual system. This question on neuroplasticity is particularly relevant in the context of rehabilitation therapies that attempt to overcome limitations or damage, through either perceptual training or retinal and cortical implants. Studies on cortical neuroplasticity have generally made the assumption that neuronal population properties and the resulting visual field maps are stable in healthy observers. Consequently, differences in the estimates of these properties between patients and healthy observers have been taken as a straightforward indication for neuroplasticity. However, recent studies imply that the modeled neuronal properties and the cortical visual maps vary substantially within healthy participants, e.g., in response to specific stimuli or under the influence of cognitive factors such as attention. Although notable advances have been made to improve the reliability of stimulus-driven approaches, the reliance on the visual input remains a challenge for the interpretability of the obtained results. Therefore, we argue that there is an important role in the study of cortical neuroplasticity for approaches that assess intracortical signal processing and circuitry models that can link visual cortex anatomy, function, and dynamics.

## 1. Introduction

Unravelling the organization of the visual cortex is fundamental for understanding the foundations of vision in health and disease. A prominent feature of this organization is the presence of a multitude of visual field maps. These maps are spatially and hierarchically organized representations of the retinal image and are often specialized to encode specific environmental visual attributes. Studying these cortical visual maps is relevant as it enables the characterization of the structure and function of the visual cortex and therefore the study of the neuroplastic capacity of the brain. With the latter, we refer to the ability of the brain to adapt its function and structure in response to either injury or to a treatment designed to recover visual function.

Over the last two decades, visual field mapping has been extensively used to infer neuronal reorganization resulting from visual field defects or neuroophthalmologic diseases. For a review, see Wandell and Smirnakis [4]. Because of its focus on the analysis of individual participants and the relative amount of detail provided, the pRF model seems ideal to study questions on neuroplasticity—at least in theory. Some of the hypotheses that can be tested with pRF mapping are as follows: are the neurons within the lesion projection zone active? Is there a displacement in position or enlargement of the pRF size during development, following a retinal or cortical lesion? Do the pRF properties change in response to monocular treatments that promote the use of the amblyopic eye, e.g., patching or blurring therapy?

Given that visual neuroplasticity is greatest during early stages of development (childhood), the characterization of the pRF properties has special relevance to determine, in vivo, the presence of atypical properties of the visual cortex during development and plasticity. In particular, changes in pRF size have been reported in a series of studies on developmental disorders. Clavagnier and colleagues measured enlarged pRF sizes in primary visual areas (V1-V3) in the cortical projection from the amblyopic eye as compared to the fellow eye [5]. Schwarzkopf and colleagues reported that individuals with autism spectrum disorder (ASD) have larger pRFs as compared to controls [6]. Anderson and colleagues found smaller pRF sizes in the early visual cortex of individuals with schizophrenia compared to controls, using a specific pRF model that takes into account the center surround structure of the RF [7].

In the case of congenital visual pathway abnormalities that affect the optic nerve crossing at the chiasm, e.g., achiasma, albinism, and hemi-hydranencephaly, several studies revealed overlapping visual fields and bilateral vertical symmetric pRF representations [8–12]. This contrasts with the case of a single patient that had her left hemisphere removed at the age of three, who did show the expected right hemifield blindness, even though she had larger representations of the central visual field in extrastriate visual maps, which was particularly apparent in area LO1 in the right hemisphere [13].

Hence, the pRF modeling approach has been applied with at least some degree of success to reveal neuroplastic changes at the level of the visual cortex. Nevertheless, in the present paper, we will briefly indicate issues with the current pRF approach as it relates to neuroplasticity and ways to improve the methods. Finally, we will argue that we should also look beyond it to fully address questions on neuroplasticity.

## 2. Limitations of Current Stimulus-Driven Approaches When Studying Neuroplasticity

*We address the question to what extent population receptive field mapping is actually a suitable tool to capture cortical plasticity. We point out various limitations. The most important one is that the assumption of the receptive field and map stability in healthy controls is largely untenable*.

The most common and straightforward manner in which the pRF approach has been applied is to compare model parameters between either two groups of participants—usually a patient group and matched controls [8, 14], or between the affected eye and the normal fellow eye, which can be done in the case of monocular developmental conditions such as amblyopia [5]. In both types of studies, it is commonly assumed that the differences in pRF estimates are caused by differences in brain organization and eye-brain connectivity of the two groups or the two eyes. However, there are various issues that complicate the interpretation of pRF differences in health and disease. A number of these limitations were recently discussed by Dumoulin and Knapen [15], and for this reason, we will only reiterate the most critical ones.

### 2.1. Changes at the Level of the Eye Limit the Use of pRF Mapping to Study Neuroplasticity in Both Ophthalmic and Neurological Diseases

Estimates of pRFs are based on the stimulus input. In numerous ophthalmic diseases, changes at the level of the eye—such as cataract or retinal lesions—strongly modify the visual input. This could be a decrease in visual acuity, contrast sensitivity, or the entire loss of vision in part of the visual field. Consequently, in many of such diseases, the stimulus-driven input to the brain will be different and usually deteriorated. In neurological conditions such as in hemianopia, retrograde degeneration of the retina [16, 17] gives rise to a similar concern. As changes in the visual input have a direct effect on the signal amplitude, straightforward differences in BOLD signal cannot be taken as an indicator of neuroplasticity or degeneration at the level of the cortex.

The retinotopic maps of healthy adults with normal or corrected to normal vision are stable over time when measured under similar environmental and cognitive factors [18, 19]. Hence, it would appear that changes in maps or population properties should be a good indication for the presence of neuroplasticity. Indeed, it was found that in patients with long-term visual impairment due to macular degeneration, the pRF of voxels representing both the scotomatic area and neighboring regions are displaced and changed in size [20].

However, there is mounting evidence that simple stimulus manipulations, e.g., masks mimicking retinal lesions, can have a large effect on the population-receptive field estimates in healthy participants. Estimated pRF properties (position shift and scaled size), similar to those in patients with retinal lesions, were observed in healthy adults in whom a visual field defect was simulated [20–22]. Comparable shifts in pRF position and scaling of pRF size were also found in an experiment that used scotopic illumination levels to examine the “rod scotoma” in the central visual field [23]. In other words, changes in visual input can mimic the consequences of lesions due to ophthalmic disease in healthy observers. This implies that observed differences in pRF properties in patients relative to controls may simply reflect normal responses to a lack in visual input rather than a reorganization of the visual cortex. Therefore, just by themselves, changes in pRF measures are insufficient to decide on the presence of neuroplasticity.

The feasibility to use pRF estimates to topographically map visual field defects in the cortex, particularly in early-stage disease, is further complicated by two aspects. First, neurons near the border of either the scotoma or the edge of the visual stimulus field may be partially stimulated. In such cases, the stimulus aperture partially activates receptive fields that belong to voxels whose pRF center would ordinarily be outside the stimulus presentation zone [21, 24]. Second, the presence or absence of a scotoma affects mostly the signal amplitude while the temporal dynamics of the modulation pattern are not affected. As pRF estimates are mostly invariant to the BOLD amplitude, the pRF model does not properly capture the effect of the scotoma. These two factors induce biases in the pRF estimates that can be wrongly interpreted as signs of neuroplasticity (see Box 2).

Nevertheless, changes in the BOLD signal may be used as an alternative assessment for nonfunctional parts of the visual system in patients that are unable to perform standard ophthalmic examinations, e.g., infants or patients with nystagmus [25–27]. However, because of the above aspects, caution is warranted when interpreting such data. Eye movements may affect the pRF estimates substantially, resulting in noisy maps and increased pRF sizes [28–30]. This is particularly relevant for developmental disorders such as amblyopia [5, 31–33]. In addition, pRF mapping is most accurate at an advanced stage of ophthalmologic disease where the visual field defects are relatively large and the scotomatic edge (i.e., the transition between healthy visual cortex and damaged visual cortex) is sharp [34, 35]. Overall, this inability to accurately detect small visual field defects implies that the sensitivity of the pRF approach is too limited to monitor the effects of slow retinal degeneration or slow cortical changes that would presumably be associated with rehabilitation therapies or other procedures to restore visual functioning.

### 2.2. Different Stimulus Properties Result in Distinct pRF Properties in Healthy Human Observers

An additional factor to be considered when interpreting pRF estimates is that the pRF represents the cumulative response across all neuronal subpopulations within a voxel. These subpopulations are selectively sensitive to spatial properties, such as orientation, color, luminance, and temporal and spatial frequencies. Hence, their activity can be driven by specific stimuli. In pRF mapping, manipulating the carrier—the stimulus aperture which drives the neuronal activity—elicits responses from a particular neuronal population. By selectively stimulating these neuronal populations, a number of recent studies have shown that compared to the standard stimulus (flickering luminance contrast checkerboard bar), pRF estimates shift in position and change their size [36–39]. These studies indicate that the recruitment of neural resources depends on the task and that there is a dependency of the retinotopic maps on the task or stimulus. This type of stimulus selectivity captures the neuronal population characteristics for features such as luminance, orientation, or words. In contrast, Welbourne and colleagues [40] found no difference in pRF estimates when using chromatic and achromatic stimuli. This implies that for color, there may be a decoupling between the pRF measurement and the underlying neuronal populations [40].

The spatial distribution of the receptive fields can also be modelled by attention. A series of studies manipulating spatial and feature-based attention found that the neuronal resources are shifted towards the attended positions [30, 41, 42].

These findings imply one of two things: (1) the topography of the visual cortex is flexible and may change in response to environmental (stimulus, task) as well as cognitive factors such as attention or (2) pRF measures are inaccurate and may change in response to spatial and cognitive factors. Either of these explanations limits the ability of the pRF approach to provide a straightforward assessment of neuroplasticity.

## 3. Improving Stimulus-Driven Approaches

*We consider various ways in which the pRF method might be improved to study neuroplasticity. Of note are models that provide information on the reliability of the pRF-estimated properties. As a further incentive, we propose a new pRF model that incorporates cortical temporal dynamics and which integrates connectivity and topography*.

Given the limitations mentioned above, this raises the question whether and how the pRF approach can be modified to render it more suitable to track neuroplastic changes. As was indicated, mimicking visual field defects can alter pRF properties in a similar manner to patients. At the minimum, this requires creating elaborated control stimulus conditions (simulations) that exactly mimic patient conditions. Unfortunately, this is often impossible to achieve. Deviations of parameter estimates in the patient group from those control values could be an indication of neuroplasticity. However, obtaining good simulations is not trivial. Thus far, the simulations that have been used have generally been quite simple, i.e., mimicking scotomas in which no light sensitivity remained—usually simulated as a region without signal modulation. However, the perceptual awareness of natural scotomas may be substantially different from that of artificial ones. For example, when the visual input is incomplete, the visual system appears to fill in any missing features (through prediction and interpolation) in order to build a stable percept. Moreover, scotomas in patients are usually more complex than simulated ones, both in their shape and their depth (reduced sensitivity). Finally, the scotoma may also change the attentional deployment by the patient, potentially affecting the estimated pRF properties [30, 41, 42].

In order to accurately measure neuronal reorganization, it is crucial to overcome the abovementioned limitations. A significant amount of work has been directed towards the development of more reliable models of retinotopic mapping. The methodological advances serve three different goals, which may be useful in studying neuroplasticity: (1) improve the reliability of the estimates using more informative pRF shapes and more complex computational models, (2) measure stimulus-selective maps, which allow to capture the reorganization of specific neuronal populations, and (3) measure spatial modulation and dynamics of neuronal populations, potentially reflecting short-term neuroplastic changes.

### 3.1. Computational and Model Advances

Computational and model advances have been made to (a) improve the pRF shape so that it better reflects the biological structure of the RF, e.g., using a difference of Gaussian model allows to account for surround suppression [43], and (b) account for nonlinearities, provide distributions of property magnitudes, and capture neuronal characteristics, such as tuning curves. Such models add new pRF features which may be important to infer functional reorganization and provide a measure of the reliability of the estimates.

A different pRF shape can be an indication of neuroplasticity. Several models have been developed to account for various possible receptive field shapes: circular symmetric difference of Gaussian (DoG) functions [43], bilateral pRF [10], elliptic shape [34], Gabor wavelet pyramids [34, 44], and compressive spatial summation [45]. Some reviews have discussed these methods in detail [15, 46]. However, the above models all assume some form of symmetry. Recently, data-driven models were developed that do not assume any a priori shape [47–49]. These model-free approaches are particularly relevant to measure the functioning of the visual system in patients, as plasticity may manifest as a differently shaped pRF without affecting its position or size. An example is that asymmetrical shapes capture best the pRF properties of any skewed distributions of RF within a voxel. However, even in these data-driven approaches, the estimated shape of the receptive fields remains dependent on the stimulus used.

**Figure 3:**
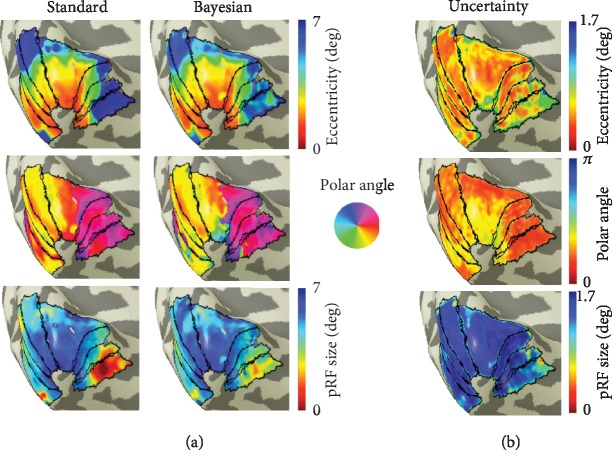
Mapping the uncertainty of model estimates: (a) maps obtained using conventional pRF mapping [3] and a custom implementation of the Monte Carlo Markov chain Bayesian pRF approach [50, 51]. Both methods result in similar visual field maps. However, the latter method also enables the estimation of the uncertainty associated with each parameter; (b) eccentricity, phase, and pRF size uncertainty maps obtained for the left hemisphere of a single healthy participant. The uncertainty maps describe how reliable each estimate is. For example, we see that the polar angle estimates for the central fovea (near fixation) are less reliable than those measured in the periphery. The uncertainty associated for each estimate was calculated as the difference between the 75% and 25% quantiles of the Bayesian Markov chain pRF distribution.

Extending the pRF model to account for more complex RF shapes will improve its explanatory power—the model can better predict the BOLD response. However, this will not remove the issue of model bias, mentioned in Box 2. In various attempts to resolve this, computational advances were made which can be categorized into four different classes. The first class comprises nonlinear pRF models, such as a compressive spatial summation model and convex optimized pRF, which substantially increases the range of shapes that the model can describe [45]. The second class is the development of Bayesian models. For each property, these models do not only estimate the best fitting value but a full posterior distribution as well [50, 51]. This serves several needs: (a) it indicates the uncertainty associated with each estimate (Figure 3). Such uncertainty maps are of particular importance when a visual field defect is present, as higher uncertainty will most likely be associated with model biases, (b) it facilitates the statistical analysis, and (c) it allows one to incorporate additional biological knowledge by providing prior information. An example of such a biologically based prior is that the density of cortical neurons is higher in the fovea than in the periphery [50, 51]. In combination, the above-referred three factors improve the interpretability of pRF estimates. The third class comprises the development of the feature-weighted receptive field (fwRF) models that allow capturing additional pRF parameters—such as neuronal tuning curves (e.g., the spatial frequency tuning)—through the combination of measured neural activity and visual features [52]. Finally, the fourth class relates to methods that allow to enhance the resolution at which we can detail RF properties. Of relevance are the approaches that allow to estimate the average single-unit RF size (suRF) [49, 53] or multiunit RF (muRF) properties that can without restriction uncover the size, position, and shape of neuronal subpopulations, also when these are fragmented and dispersed in visual space [49, 53].

### 3.2. Models of Perception: Spatial Modulation and Dynamics

Specific models have been developed to capture short-term plasticity. Such models take into account cognitive and/or perceptual factors such as attention [30, 54] or crowding [55, 56] to understand changes in observed spatial properties or perception. Recently, Dumoulin and Knapen proposed a more complex pRF model that relates pRF changes to the underlying neural mechanisms [15]. This very general model allows modeling and predicting dynamic changes that result from changes in the visual input. In particular, they proposed an extension of the pRF model to account for multiple neural subpopulations responding to different properties of the stimulus. Their expectation is that this will enable unravelling of the different sources of pRF plasticity.

Although there have been significant improvements in pRF models which may be able to aid in charting neuroplastic changes, in our view, this is still insufficient. There are still many constraints to be addressed, in particular, the fact that a voxel may contain a mixture of neurons with spatially distinct receptive fields. This is particularly relevant in developmental disorders such as albinism and achiasma [9, 10] or for voxels located in sulci. In those cases, the measured pRF properties will either represent the strongest contributing RF or be erroneously large.

In our view, the neuronal spatiotemporal dynamics can be better captured if we would take into account the interactions with nearby linked populations. The connectivity-weighted pRF, described next, is a first attempt to integrate models of cortical organization with cortical connectivity. This further encourages the development of new models that integrate stimulus- and cortex-referred methods.

### 3.3. The Connectivity-Weighted pRF Integrates Cortical Organization and Connectivity

Current analytical approaches to track retinotopic changes are voxel based. This limits their accuracy, as the visual system is dynamic and the activity of one population of neurons is influenced by nearby connected populations. Ideally, a more complete model should reflect the balance between inhibitory and excitatory processes and account for various cortico-cortical interactions.

Here—as an example of such a model—we propose a stimulus-driven pRF model, in which the estimated parameters, *pRF*_*j*_, depend upon the unique activity of the neuronal population *pRFu*_*j*_ and the activity of interacting cortical neuronal populations, weighted by the strength of their connections, *C*_*jk*_. Note that *e*_*j*_ is the error associated with voxel *j*.

(1) $$\begin{array}{c} {pRF}_{j}={pRFu}_{j}*\left(\underset{k\neq j}{\sum }C_{jk}*{pRF}_{k}\right)+e_{j}. \end{array}$$

Depending on the goal of the study and the design of the experiment, the connectivity (*C*) can be based either on the structure (anatomically connected neighbors), on function (neuronal populations which exhibit specific correlated activity during the resting state), or on effective connectivity [57]. Here, we treat it as effective connectivity given that it accounts for dynamic interactions and the model of coupling between neuronal populations.

Such a model can describe the spatiotemporal dynamics of neuronal populations. It is sensitive to the recurrent flow of synchronized activity between connected neurons. Using such a connectivity-weighted model, we may—in the future—assess brain plasticity based on both structural reorganization and functional reorganization.

## 4. Cortical Circuitry Models Look beyond the Stimulus

*We suggest that models that can be estimated without requiring visual stimulation, which we refer to as cortical circuitry models (CCM), may be highly suitable to measure cortical reorganization. While not without potential pitfalls themselves, such approaches avoid many of the complications associated with the stimulus-driven pRF approach. Additionally, we indicate various other avenues that may improve our ability to quantitatively assess neuroplastic changes in the visual cortex*.

### 4.1. Studying Neuroplasticity Using Intrinsic Signals and Cortical Circuitry Models

The fMRI signal is a mixture of stimulus-specific and intrinsic signals [57, 58]. As a result, it is plausible to assume that intrinsic generated signals may influence stimulus-driven signals [57, 58]. Therefore, the study of brain plasticity may be ameliorated and/or complemented if the dependence on stimuli is reduced. For this reason, estimates based on intrinsic signals rather than task responses are potentially a very suitable source of information on the presence or absence of cortical plasticity. Intrinsic signals are commonly obtained in a “resting-state” condition in which participants are not required to do anything in particular and usually have their eyes closed. Resting-state fMRI signal fluctuations have been shown to correlate with anatomically and functionally connected areas of the brain. In particular, specialized networks have been found in cortical and subcortical areas in sensory systems [59–64]. Based on resting-state data, CCMs can be used to infer the integration of feedback and feedforward information [65]. However, one important limitation is that currently, the directionality of information flow cannot be directly inferred from the BOLD signal. Therefore, primarily because of the limited temporal resolution of fMRI, it remains to be determined whether CCMs can be used to assess this aspect.

Nonetheless, CCMs have the potential to capture the effects of structural reorganization and can inform about which neural circuits have the potential to reorganize and which are stable. An example of this type of model is the connective field (CF) model, which applies the notion of a receptive field to cortico-cortical connections [66]. Another example is the connectopic model which combines voxel-wise connectivity “fingerprints” with spatial statistical inference to detail multiple overlapping connection topographies (connectopies) in the human brain [66, 67]. Ultimately, in our view, it will be essential to combine retinotopic and neural circuitry models, such that their combination can be used to fully describe the dynamics of the visual cortex [68]. To accomplish this, models will have to be developed that can capture the (dynamic) adaptation of feedback, feedforward, and lateral connections in the functional networks underlying visual processing and cognition. Such models may be implemented by calculating the correlation between neuronal populations taking time lags into account or by using CCM to describe connections across cortical layers (see also below).

### 4.2. The Connective Field Defines a Receptive Field in Cortical Surface Space

Connective field (CF) modeling predicts the neuronal activity in a target area (e.g., V2) based on the activity in a source area (e.g., V1). In a similar way that a neuron has a preferred location and size in visual space (its receptive field), it also will have a preferred location and size on the cortical surface of a region that it is connected with [65, 66, 68]. Based on retinotopic mapping, the visual field coordinates of the target area can be inferred from the preferred locations in the source region. In this way, the connective field—when combined with pRF mapping—can link a CF's position in cortical surface space also to a position in visual space. The connective field model is briefly described in Box 3.

There are several advantages of CCMs when compared to pRF models. First, the ability to assess and compare the fine-grained topographic organization of cortical areas promotes the comparison of connectivity patterns between groups of participants with different health conditions and between experimental conditions [67, 70]. Second, CCMs can even be applied to data that was acquired in the absence of any sensory input, enabling the reconstruction of visuotopic maps even in the absence of a stimulus and in blind people. Several studies have shown that cortical connectivity during the resting state reflects the visuotopic organization of the visual cortex [65, 67, 70–73]. A comparison between stimulus-driven and resting-state CCMs may also convey information on the influence of retinal waves and prior visual experience in the cortical circuitry. For example, larger CF sizes were measured with visual stimulation when compared to the resting state [65, 73, 74]. Third, CCMs provide insight into the anatomical and functional neuronal circuitry that enables the visual system to integrate information across different cortical areas. They can reveal the presence or absence of a change therein following a disease [74–76]. Fourth, CCMs, in particular when assessed in the resting state, are less affected by various intrinsic and extrinsic factors such as the type of task and stimulus [37–39], patient performance, optical properties and health condition of the eye [77], or stimulus-related model-fitting biases [22, 77].

Despite these important advantages, the current CCM approaches also have their limitations. First, the reliability of CCM parameters, such as the CF size, is affected by the signal-to-noise ratio. Fortunately, the signal-to-noise ratio does not introduce a systematic bias in the estimated parameters [74–76]. Second, the current iteration of CCM models does not capture causal interactions between different cortical visual areas. Third, like pRF estimates, it is likely that the accuracy of the CCM-related estimates depends on the spatial and temporal resolution, the distortion and spatial spread of the BOLD signal, and the distribution of dural venous sinuses and vessel artifacts. Fourth, although there is no need for stimulus-driven signals, resting state signals—and thus also any estimated CCM properties—are influenced by the environmental conditions under which they were acquired. Factors such as eye movements and exterior luminance may also influence estimates. These limitations demonstrate that although the CCM approach seems suitable to infer the presence or absence of plasticity by associating connectivity strength with cortical degeneration [75], it still requires careful experimentation as well.

Some of the above limitations have recently been addressed. For example, global search algorithms that help to avoid local minima have also been applied to CCMs [74, 75]. Furthermore, new data-driven methods are able to measure multiple and even overlapping connectopies [67]. Although, currently establishing these connectopic maps requires a very large number of participants, they hold a promise of being able to reveal cortical and network reorganization and plasticity one day [67].

### 4.3. Cortical Circuitry Models in Ophthalmic or Neurological Diseases

The development of CCMs is a sequel to the classical pRF mapping. Hence, the available literature is still relatively small. Nonetheless, the existing studies give a good impression of the possible applications and the type of information that these models can provide.

At this point in time, in particular, the CF modeling approach has been applied in several ophthalmic disorders, in which visual perception was either impaired or completely absent. A study by Haak and colleagues found that in macular degeneration, long-term deprivation of visual input had not affected the underlying cortical circuitry [75]. This suggests that the visual cortex retains the ability to process visual information. In principle, following the restoration of visual input, i.e., via retinal implants, such patients may thus recuperate from vision loss. Papanikolaou and collaborators applied CF modeling to study the organization of area hV5/MT+ in five patients with large visual field defects resulting from either early visual areas or optic radiation lesions [76]. They showed that in three of the five subjects, the CFs between areas V1 and hV5/MT+ covered visual field locations that overlapped with the scotoma. This indicates that activity in the lesion projection zone in hV5/MT+ may originate from spared V1. Bock and collaborators applied the CF model to resting-state BOLD data acquired from normally sighted, early blind, and monocular patients in which one of the eyes had failed to develop [74]. All subjects showed retinotopic organization between V1 and V2/V3. Butt and colleagues studied the cortical circuitry of the visual cortex in blind observers and compared this to that of sighted controls [70, 74]. They found a very minute change in the pattern of fine-scale striate correlations between hemispheres, in contrast to the highly similar connectivity pattern within hemisphere. They concluded that the cortical connections within a region (which can be a hemisphere) are independent of visual experience. The above-cited studies show that, in general, the visuotopic organization of the cortical circuitry is maintained even after prolonged visual deprivation or blindness, supporting that the plasticity of the adult visual brain is limited (see Wandell and Smirnakis for a similar conclusion based on stimulus-driven mapping [4]). Moreover, these studies suggest that CCMs may be able to capture the integrity of cortical connections using both stimulus-driven and resting-state data. This encourages the development of new CCMs that can be applied to study how connected neurons in different layers and columns interact.

### 4.4. Mesoscale Plasticity: Layer- and Column-Based Cortical Circuitry Models

Measuring cortical reorganization at a finer scale might reveal changes that are invisible or masked at a coarser scale. With the recent advance in ultra-high field functional MRI, the tools to examine the human brain at a mesoscale in vivo have become available. This enables assessing the presence of cortical reorganization across cortical depth to measure the flow of information across different cortical laminae—in particular feedback and lateral inputs—and to infer the microcortical circuits by studying their columnar organization.

Many of the opportunities and challenges in visual neuroscience provided by increases in MRI field strength have been described in a recent review, to which we refer [78]. With respect to the topic of neuroplasticity, a study that showed that pRF in the input (middle) layer have a smaller RF than those in superficial and deeper intracortical layers is of particular interest [79]. Although this study provides hints about cortical organization, it exclusively relied on stimulus-based modelling and thus does not truly inform about the underlying circuitry. In order to bridge this gap, we propose that the application of CCM-like approaches to study short-range connections at laminar and columnar levels is warranted.

The development of methods that reflect the mesoscale circuitry should be able to answer various outstanding critical questions in visual neuroscience and contribute with new fundamental and clinically relevant insights into cortical functioning and neuroplasticity. For example, following a visual field defect, is the input/feedforward layer the one that is most affected? Do neurons in the upper and deepest layers of the lesion projection zone establish new connections to healthy neurons in the input layer? At what level of cortical processing do feedback and feedforward signals modulate our conscious percepts? Are putative overlapping representations in ventral areas [38] perhaps encoded in distinct layers of the visual cortex?

## 5. Conclusion

In this paper, we discussed (a) the role of pRF mapping to cortically characterize visual areas and extrinsic and intrinsic factors that influence the pRF estimates, (b) methodological advances in retinotopic and connectopic mapping, and (c) stimulus-driven and cortical circuitry models that can link visual cortex organization, dynamics, and plasticity.

Although we fully acknowledge the important contribution of pRF mapping towards understanding the structure and functioning of the visual cortex, we strongly argue against a “blind” reliance on this technique when studying neuroplasticity. The degree to which a change in signal amplitude or pRF measurements—by themselves—reflects that cortical reorganization remains to be determined: even in the presence of a presumed stable cortical organization in healthy participants, different pRF estimates may be elicited due to a change in the task at hand, cognitive factors, and the type of stimulus used. For this reason, we have stressed that prior to deciding that pRF changes are the result of reorganization, one has to exclude that these are due to different inputs, (implicit) task conditions, or cognitive demands.

To improve the reliability of retinotopic mapping, more complex models and computational approaches have been developed with a noticeable trend to move from stimulus-driven to data-driven techniques. These efforts have resulted in a multitude of new methods. Their specific use depends upon the goal of the study and the neuronal population of interest. Nevertheless, although these newer techniques provide clear improvements, they potentially retain the issues associated with stimulus-driven approaches. Therefore, we argue in favor of also considering alternative techniques to study brain plasticity, in particular ones that directly assess the neural circuitry rather than stimulus-driven responses to estimate the extent of neuronal reorganization. As an exemplary incentive, we propose a model that combines connectivity with spatial sampling. In theory, such a model will not only inform about the spatial sampling but also about interactions between the linked neuronal populations. Finally, we encourage the development and application of models to capture the plasticity of layer-based circuitry at the mesoscale.

## Figures and Tables

**Figure 1:**
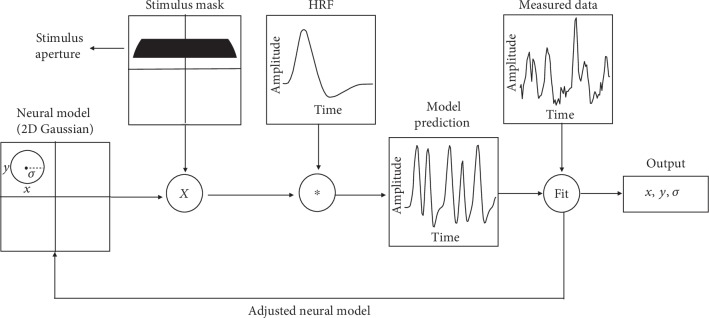
The population receptive field (pRF) modeling procedure. A pRF model describes, per voxel, the estimated pRF properties position (*x*, *y*) and size (*σ*). A voxel's response to the stimulus is calculated as the overlap between the stimulus mask (the binary image of the stimulus aperture: a moving bar) at each time point and the receptive field model. Following this, the delay in hemodynamic response is accounted for by convolving the predicted time courses with the hemodynamic response function. Finally, the pRF model parameters are adjusted for each voxel to minimize the difference between the prediction and the measured BOLD signal. The best fitting parameters are the output of the analysis. Figure adapted from Dumoulin and Wandell [3].

**Figure 2:**
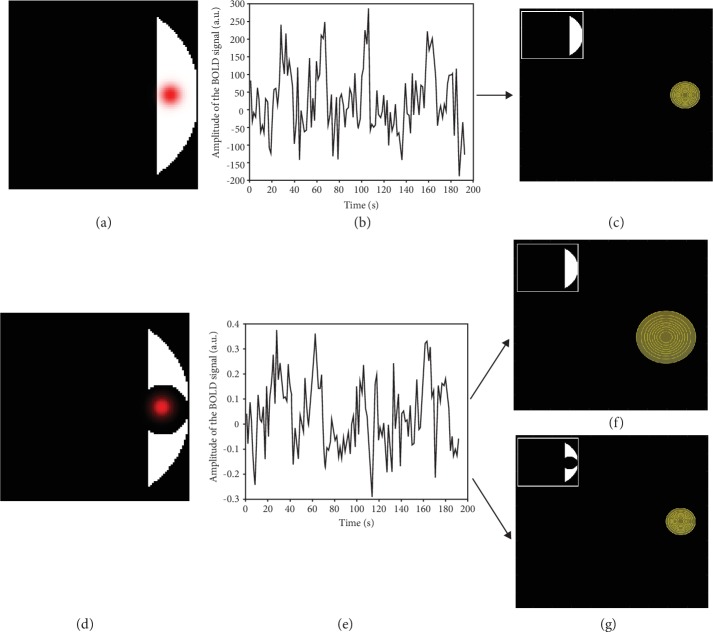
Simulated pRF time series and the associated estimated pRF properties: (a) simulation of a pRF (red) located at a specific region of the visual field (*x* = 5, *y* = 0) and with a size of *σ* = 0.5 deg assuming normal vision (i.e., no scotoma); (b) simulated fMRI response given the retinotopic stimulus (a) modelled with added noise (signal to noise ratio of 1 : 1); (c) estimated pRF using the normal vision simulated time series (b). The mask used in the pRF model is presented in the upper left corner. The estimated properties were identical to the simulated ones: *x* = 5, *y* = 0, *σ* = 0.5 deg, and a variance explained of 0.46. (d, e) are analogues to (a, b), but for a simulated pRF located in the lesion projection zone (thus inside the simulated scotoma); (f) estimated pRF based of the scotoma simulated time series (e) using a mask that assumes normal vision. The estimated pRF shifted in position and increased in size (estimated position shifted towards *x* = 4 and *y* = −1 and the size was enlarged, *σ* = 1 deg). The variance explained obtained was 0.45; (f) estimated pRF based of the scotoma simulated time series (e) and taking into account the lesion by using a mask that includes the scotoma (upper left corner). The estimated pRF properties are now again identical to the simulated ones (*x* = 5, *y* = 0, *σ* = 0.5 deg, and variance explained = 0.44).

**Figure 4:**
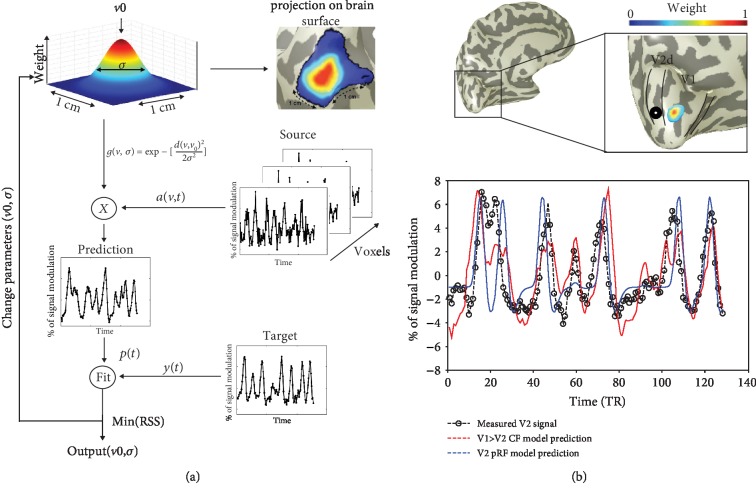
(a) CF pipeline as described by Haak and colleagues [66]. The model comprises two steps: (1) predict the fMRI response, *p*(*t*), by multiplying the CF model *g*(*v*0, *σ*) with the measured source fMRI signal *a*(*v*, *t*), and (2) the CF position (*v*) and size (*σ*) are estimated by varying parameters and selecting the best fit between the predicted time series and the measured BOLD signal *y*(*t*). Then this procedure is repeated for every voxel in the target region. (b) The V2 response is predicted based on the pRF (stimulus-driven, in blue) and connective field (cortical-driven, in red) model. The color map on the brain shows the V1>V2 CF model weights for a specific voxel.

**Box 1:**
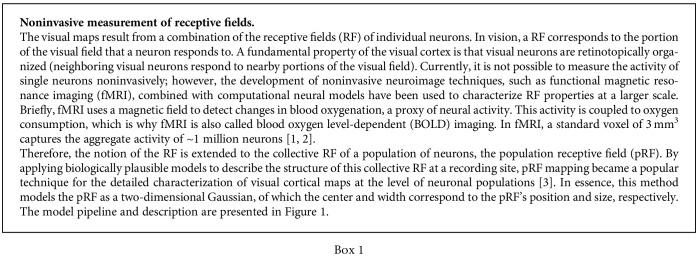


**Box 2:**
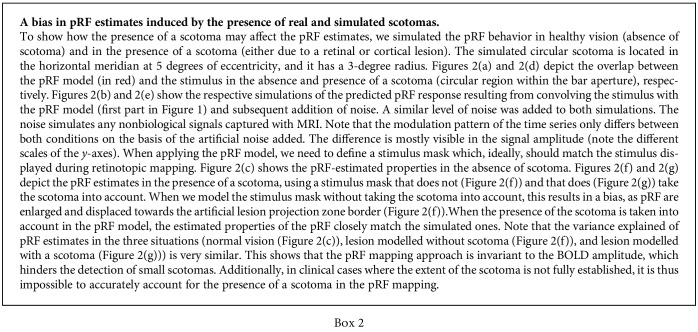


**Box 3:**